# Screen-viewing among preschoolers in childcare: a systematic review

**DOI:** 10.1186/1471-2431-14-205

**Published:** 2014-08-16

**Authors:** Leigh M Vanderloo

**Affiliations:** 1 Health and Rehabilitation Sciences, Western University, 1201 Western Rd., Elborn College Rm 2585, London, ON N6G 1H1, Canada

**Keywords:** Screen-viewing, Preschool-aged children, Childcare, Sedentary behavior

## Abstract

**Background:**

Screen-viewing is one of the most common sedentary behaviors among preschoolers. Despite the high prevalence of sedentary behaviors in childcare, little research exists on the context and/or type of activities that account for these particular behaviors. Accordingly, if the amount of screen-viewing accumulated by preschoolers in childcare is not considered, researchers may be underestimating total screen time among this population, as only a portion of their day is being captured (i.e., the home environment). This systematic review provides a synthesis of research on the levels of screen-viewing among preschool-aged children (2.5-5 years) attending childcare (i.e., centre- and home-based childcare). This review also examined the correlates of screen-viewing among preschoolers in this setting. To provide additional contextual information, *availability of screen activities* was used to help ameliorate the understanding of preschoolers’ screen-viewing behaviors in childcare.

**Methods:**

Twelve electronic databases were searched to retrieve relevant articles for inclusion (dating from 2000 onwards). Additional studies were identified via manual searching techniques (i.e., hand searching and citation tracking). Only English, published peer-reviewed articles that examined preschoolers’ screen-viewing behaviors in childcare (i.e., rates of screen-viewing and access to/opportunities for related activities) were included. No restrictions to study design were applied.

**Results:**

Seventeen international studies (4 experimental; 12 cross-sectional; 1 mixed-methods) published between 2004 and 2014 were examined. Of those, eight studies reported rates of screen-viewing and found that preschoolers spent approximately 0.1 to 1.3 hrs/day and 1.8 to 2.4 hrs/day engaged in this behavior in center- and home-based childcare, respectively. High staff education (negative association) and type of childcare arrangement (notably, home-based childcare in comparison to center-based childcare; positive association) were identified as two correlates in relation to preschoolers’ screen-viewing in childcare. Nine studies spoke to the availability of screen-viewing activities in childcare, and found the childcare environment to be conducive to this behavior.

**Conclusions:**

Despite some variability, preschoolers appear to engage in somewhat high levels of screen-viewing while in childcare, particularly within home-based facilities. This paper also highlighted the conduciveness of the childcare environment with regard to screen-viewing among preschoolers. Additional exploration into the correlates of screen-viewing in childcare is required. (PROSPORO registration: CRD42013005552).

## Background

One of the most common sedentary activities in which preschoolers participate is screen-viewing [1]. Often a proxy measure for sedentary activity, [2] screen-viewing encompasses a variety of activities, including: television, DVDs/VHS, video games, computers, and smartphones. The omnipresence of screens in children’s lives is not surprising, given the drastic shift in device availability, program development, and marketing efforts over the past two decades [3,4]. International statistics indicate children in Canada (i.e., 3–4 years old), [5] the United States (i.e., 4–7 years), [6,7] and Australia (i.e., 2–6 years) [8,9] are spending between 1.5 to 7.0 hours daily in screen-viewing activities. Even more disturbing is the recognition that the length of time children spend watching screens exceeds that of any other single activity in which they typically engage after sleeping [10-12]. Excessive screen-viewing is associated with a multitude of ramifications among preschool- and school-aged children, including: high blood pressure, [13] obesity [14-17], behavioral issues, [18] academic issues, [19] irregular sleep patterns, [20] and prevalent feelings of sadness and boredom [7]. Given the early years play a fundamental role in the development of health-related behaviors, including screen-viewing and physical activity, [21] early intervention is required to prevent excessive sedentary behaviors from carrying forward long-term [22,23].

Screen-viewing among children remains a global health concern. Recent research which aimed to solicit international consensus on research priorities concerning physical activity and sedentary behaviors among children and youth, [24] ranked screen-time reduction as number 9 of 29 items. A number of guidelines from various countries have been created in response to the growing rates of sedentary behaviors among young children; the American Academy of Pediatrics stipulates children’s (i.e., over 2 years) screen-viewing should be limited to a maximum of 2 hours per day [25]. Canada’s (children aged 1–4 years) and Australia’s (children aged 2–5 years) respective guidelines encourage limiting children’s screen-viewing to 1 hour per day [26,27].

While investigations exploring screen-viewing among young children in the home environment, [28] as well as for children under 3 years, [29] have been carried out; none have specifically examined the correlates of screen-viewing among preschoolers in childcare. Carson and Janssen echo this sentiment by stressing the importance of examining other institutions’ (i.e., outside the home) associations with screen time among young children [30]. In order to reduce screen time in childcare, it is imperative that the correlates which influence this sedentary behavior be identified and understood more clearly before change can occur. A deeper comprehension of such factors is required to help inform early childhood education and developmental practices.

Despite the high prevalence of sedentary behaviors among preschoolers in childcare, [31-33] little research exists on the context and/or type of activities that account for these particular behaviors. In fact, Ward and colleagues underlined screen-time as a significant area of focus with regards to obesity prevention efforts in early childhood settings [34]. A review by Christakis found that the majority of estimates of young children’s screen-time have failed to include viewing that occurs in non-parental caregiving settings. Accordingly, if the amount of screen-viewing accumulated by preschoolers in childcare is not considered, researchers may be underestimating total screen time among this population, as only a portion of their day is being captured (i.e., the home environment). Although the literature highlights the potential of the childcare environment to provide preschoolers with a number of opportunities to learn and adopt healthy behaviors (including those related to screen-viewing), [35] this setting is posited as an important venue of focus; [36] which is especially true considering the magnitude of young children enrolled in this setting [37-39] as well as the number of hours spent in care [40-42].

The development of a synthesized document estimating the amount of time this cohort spends in screen-viewing activities in childcare, as well as ascertaining whether this behavior is in fact problematic in an environment typically considered sedentary in nature, is warranted [33,41,43]. Additionally, little is known concerning the factors within the childcare environment that influence screen-viewing. Given the many negative health outcomes associated with excessive screen-viewing, [7,13,18-20,36,44] the creation of this document would certainly provide additional insight into this body of research.

The current study sought to systematically review and synthesize all relevant literature to assess preschoolers’ screen-viewing time in childcare (i.e., center- and home-based). A secondary objective was to examine the correlates of screen-viewing among preschoolers in childcare. To provide additional contextual information, *availability of screen activities* (a commonly used construct), was examined to supplement our understanding of preschoolers’ screen-time and behaviors while in care, and has been correlated with increased screen-time among children [16,28].

## Methods

As a means of optimizing the rigor, clarity, and transparency of the current review’s findings, the PRISMA statement for systematic reviews was utilized [45,46]. This review is registered with PROSPERO (registration no. CRD42013005552).

### Eligibility criteria

Published, peer-reviewed, English-language studies were included if there was a quantitative measurement of screen-viewing in childcare (e.g., center- and home-based childcare, family childcare homes, daycare, nursery school, preschool setting, etc.). All methods of assessing screen-time (e.g., observation, self-/proxy-report) were considered. Given that *combined* screen-time was of interest (i.e., across multiple screens), all literature pertaining to time preschoolers spent utilizing or engaging with various screens (i.e., television, computer, video games, smartphones, DVD/VHS) were included. *Availability of screen-viewing activities* was examined and included as well in order to gain a deeper understanding of the screen-viewing environment among preschoolers in childcare. Only studies focusing on children between the ages of 2.5 and 5 years were included. In cases of intervention and cohort studies, only baseline measurements were acknowledged. Given the low number of available primary research articles on the proposed review topic, all study designs and quality of evidence were considered.

### Search strategy and study selection

Using a comprehensive search strategy^a^, 12 electronic databases were searched: Embase, CINAHL, PubMed, ProQuest Allied Health and Nursing, SPORTDiscus, Medline, PyschInfo, ProQuest Dissertations and Theses, ProQuest Educational Journals, Scopus, Physical Education Index, and Sociological Abstracts. See Table 1 for one example of a search strategy used. The search frame of these electronic record searches dated from 2000 onwards; the final database search was run March 8, 2014. All retrieved articles were exported to Reference Manager software (version 12) and duplicates were removed manually from the database. Each entry in the database was assigned a unique identification number.

**Table 1:** A sample search strategy utilized for the present review (EMBASE)

** #**	**Searches**	** Results**
1	preschool child/	514746
2	preschool*.mp.	521258
3	“early years”.mp.	3276
4	“early childhood”.mp.	22013
5	“preschool-aged children”.mp.	1006
6	“inactivity”.mp.	12005
7	“sedentary activity”.mp.	381
8	“sedentary lifestyle”.mp. or exp sedentary lifestyle/	5478
9	“physical inactivity”.mp.	4922
10	“sitting”.mp. or exp sitting/	26772
11	“movement”.mp. or exp “movement (physiology)”/	438846
12	“inaction”.mp.	557
13	“inactiveness”.mp.	19
14	“exercise”.mp. or exp exercise/	339685
15	“physical activity”.mp. or exp physical activity/	258688
16	motor activity.mp. or exp motor activity/	376722
17	“physical fitness”.mp. or exp fitness/	30887
18	“screen-viewing”.mp.	46
19	“screen viewing“.mp.	46
20	“tv“.mp.	11694
21	“television”.mp. or exp television/	21878
22	“video games”.mp. or exp recreation/	41745
23	exp computer/ or “computer”.mp.	1057438
24	“mobile phone”.mp. or exp mobile phone/	7109
25	“cell phone”.mp.	1084
26	“PDA”.mp.	8288
27	“smartboards”.mp.	0
28	“screen-media”.mp.	40
29	“screen media”.mp.	40
30	“computer games”.mp.	572
31	“tablets”.mp.	43489
32	“computer tablets”.mp.	8
33	“iPad”.mp.	505
34	“iPod”.mp.	251
35	“MP3 players”.mp. or exp MP3 player/	148
36	“electronic games”.mp.	94
37	“movies”.mp.	2308
38	“DVD”.mp.	1398
39	“smartphones”.mp.	362
40	“internet”.mp. or exp Internet/	83349
41	“multiscreen viewing”.mp.	2
42	“multi-screen viewing”.mp.	2
43	exp television viewing/	1160
44	“wii”.mp.	506
45	“videocassette”.mp.	76
46	“videotape”.mp. or exp videotape/	6009
47	“screen-based entertainment”.mp.	8
48	“screen based entertainment”.mp.	8
49	“media entertainment”.mp.	9
50	“visual entertainment”.mp.	1
51	“viewing habits”.mp.	88
52	“nintendo DS”.mp.	9
53	“interactive media”.mp.	124
54	“handheld media”.mp.	1
55	“handheld computer”.mp.	224
56	“gameboy”.mp.	5
57	exp technology/ or “technology”.mp.	345294
58	18 or 19 or 20 or 21 or 22 or 23 or 24 or 25 or 26 or 27 or 28 or 29 or 30 or 31 or 32 or 33 or 34 or 35 or 36 or 37 or 38 or 39 or 40 or 41 or 42 or 43 or 44 or 45 or 46 or 47 or 48 or 49 or 50 or 51 or 52 or 53 or 54 or 55 or 56 or 57	1536268
59	6 or 7 or 8 or 9 or 10 or 11 or 12 or 13 or 14 or 15 or 16 or 17	1193663
60	1 or 2 or 3 or 4 or 5	539762
61	58 and 59 and 60	2156
62	“active gaming”.mp.	12
63	“active games”.mp.	37
64	“childcare”.mp. or exp child care/	50827
65	“child care”.mp.	33633
66	“daycare”.mp. or exp day care/	10037
67	“day care”.mp.	12935
68	“nursery school”.mp. or exp nursery school/	1982
69	“nurseries”.mp.	2514
70	18 or 19 or 20 or 21 or 22 or 23 or 24 or 25 or 26 or 27 or 28 or 29 or 30 or 32 or 33 or 34 or 35 or 36 or 37 or 38 or 39 or 40 or 41 or 42 or 43 or 44 or 45 or 46 or 47 or 48 or 49 or 50 or 51 or 52 or 53 or 54 or 55 or 56 or 62 or 63	1200783
71	64 or 65 or 66 or 67 or 68 or 69	68560
72	59 and 60 and 70 and 71	131

*Note*. The asterix symbol (*) was used as a Boolean Operator (specially, a wildcard) to search for all variations of a particular word.

All titles and abstracts of potentially relevant articles were screened using a Title and Abstract Screening Form developed for this review. This screening form was reviewed by a second researcher to ensure the inclusion of appropriate eligibility standards. Articles meeting the initial screening parameters were retrieved in-full. A similar review method was used to appraise the full-text articles; a Full-Text Screening Form was created and applied. A second, independent reviewer screened the titles/abstracts as well as full-text articles to confirm the author’s extracted findings. More specifically, the reviewers compared their results to agree on a list of articles that met the inclusion criteria and would be appropriate to retrieve a full-text copy. Subsequently, each full-text document was read independently by the second reviewer to assess the appropriateness for inclusion, and again compared for consensus. Any discrepancies were discussed as a pair. Six authors were contacted for further information. Five replied, and two provided additional clarification regarding their respective studies’ findings [47,48]. A final set of articles was agreed upon by both researchers.

The reference lists of all articles pulled for full-text screening were also reviewed. Additionally, the table of contents of five journals (which appeared to publish a number of relevant articles; i.e., the *International Journal of Behavioral Nutrition and Physical Activity*, the *Journal of Physical Activity and Health*, *Preventive Medicine*, *Pediatrics*, and *Early Childhood Research Quarterly*) were searched manually from 2000 to present. Lastly, in an effort to be exhaustive, the advance publication or *in press* sections of 13 physical activity and childcare-related periodicals were reviewed to ensure all relevant literature was retrieved (i.e., *Journal of Physical Activity and Health; Pediatrics; International Journal of Behavioral Nutrition and Physical Activity; Preventive Medicine; American Journal of Preventive Medicine; Applied Physiology, Nutrition, and Metabolism; Journal of Sport Sciences; Research Quarterly for Exercise Science; Medicine & Science in Sport & Exercise; Early Childhood Research Quarterly; Pediatric Exercise Science; Journal of American Medical Association Pediatrics [*formerly *Archives of Pediatric and Adolescent Medicine*]). All unique articles found via these search methods underwent the aforementioned screening process.

### Quality assessment of literature

A modified version of Downs’ and Black’s checklist for quality assessment was used throughout this procedure [49]. Comparable to previous approaches, [29] only 10 of 27 items from this document were considered as they were the most relevant to this review (i.e., *clear aim/hypothesis articulated?; are outcomes clearly described in the Introduction and Methods sections?; participant characteristics provided?; main findings clearly described?; are estimates of random variability supplied?; are actual probability values reported?; were invited participants representative of the population from which they were recruited?; were the participants willing to participate representative of the population from which they were recruited?; were appropriate statistical tests used to assess the main outcomes?; were valid/reliable measures used to assess the main outcomes?*) [49]. Although the aforementioned factors were all considered while compiling the studies, all qualities of evidence were included in light of the limited research conducted on this particular topic.

### Data extraction

Study characteristics were included in a standardized extraction table (see Table 2). Findings from each study relating to rates of screen-viewing and the availability of this particular activity in childcare were also extracted and collated. Influential factors (or correlates) to screen-viewing in childcare were identified and summarized where available (i.e., direction of association, percent association, strength of consistency, etc.).

**Table 2:** Characteristics of included studies which examined rates of screen-viewing and/or screen-viewing opportunities in childcare

**Authors**	**Country**	**Design**	**Sample**	**Study purpose**	**Method of assessing screen-viewing**	**Availability of screen-viewing activities**	**Rates of screen-viewing**
▪ Bacigalupa (2005) [47]	▪ United States	▪ Mixed-methods	▪ 1 home-based childcare facility	▪ Three-fold:	▪ Field notes (direct observation)		▪ Each child permitted 18 minutes of video games/day:
			▪ 6 preschoolers (mean age = ~5 years^†∇^; 50% male)	1. Examine video game use by young children			- Children sat and watched the others play (6 children x 18mins = 108 mins/day or 1.8 hrs/day)
				2. Explore the nature of children’s interactions during video game use			- Could “earn” extra minutes for good behavior
				3. Assess video game usage within the home childcare environment			
▪ Brown et al. (2009) [43]	▪ United States	▪ Cross-sectional	▪ 24 center-based childcare facilities	▪ Two-fold:	▪ OSRAC-P (direct observation)		▪ 0.15 hrs/day (or 8.92 mins/day) per child
			▪ 476 preschoolers (mean age = 4.2 years [*SD* = 0.7]; 50% male)	1. Describe the PA behaviors and the accompanying environmental/social events of preschoolers in childcare			- 2% was in light PA
				1. Examine which conditions were predictors of MVPA and total PA			- 98% was sedentary
▪ Christakis & Garrison (2009) [50]	▪ United States	▪ Cross-Sectional	▪ 168 childcare facilities (84 home-based, 74 center-based)	▪ Two-fold:	▪ Telephone survey (proxy-report measure)		▪ Mean (*SD*) television viewing across *all* participating facilities:
			▪ Preschoolers’ age range = 3–5 years	1. Investigate characteristics of programs that predict screen-viewing			- Home-based: 2.4 hrs/day (1.8)
				2. Quantify television viewing in childcare settings			- Center-based: 0.4 hrs/day (0.9)
							▪ Mean (*SD*) television viewing across facilities that reported *any* screen-use in care:
							- Home-based: 3.4 hrs/day (2.8)
							- Center-based: 1.2 hrs/day (1.3)
							▪ Preschoolers in home-based childcare engaged in significantly more television than those in center-based care (*p <* .001)
							▪ > 90% of childcare facilities reported television being for *educational* or *educational and entertainment* purposes
▪ Christakis et al. (2006) [11]	▪ United States	▪ Cross-sectional	▪ 2,672 childcare facilities (583 home-based; 2,089 center-based)	▪ Two-fold:	▪ Survey (proxy-report measure)		▪ Mean hours of daily television viewing:
			▪ Preschoolers’ age range = 3–5 years	1. Describe the amount and frequency of television viewing among preschoolers in childcare			- Home-based: 1.39 hrs/day^∇^
				2. Explore predictors of television viewing in the childcare setting			- Center-based: 0.36 hrs/day^∇^
							▪ Preschoolers in home-based childcare watched ~4x more television than those in center-based care
▪ Dowda et al. (2004) [51]	▪ United States	▪ Cross-sectional	▪ 9 center-based childcare facilities:	▪ Determine if levels of MVPA among preschoolers varied with differences in policies/practices, and overall quality of childcare facilities	▪ ECERS-R (direct observation)		▪ 3 PAP centers, < 45 mins/day (or < 0.75 hrs/day):
			- 3 PAP centers (83 children)				- 5.7% (*SD* = 1.6) was in MVPA
			- 6 NPAP centers (183 children)				- 57.5% (*SD* = 4.8) was sedentary
			▪ 266 preschoolers (mean age = 4 years^∇^; 47% males)				▪ 6 NPAP centers, ≥ 45 mins/day (or ≥ 0.75 hrs/day):
							- 7.1% (*SD* = 1.1) was in MVPA
							- 56.7% (*SD* = 3.4) was sedentary
▪ Finch et al. (2012) [52]	▪ Australia	▪ Quasi-experimental (intervention, 2-arm)	▪ 484 center-based childcare facilities (275 intervention, 209 control)	▪ Describe impact of an intervention tasked with increasing the adoption of PA-promoting policies/practices in center-based childcare	▪ Telephone survey (proxy-report measure)	▪ Prevalence of PA policies/practices in centers (at baseline):	
			▪ Preschoolers’ age range = 3–5 years			- 45-60%: *policy that limits screen time*	
						- 17-23%: *children are allowed to watch screens less than once per week*	
▪ Finch et al. (2014) [53]	▪ Australia	▪ Cluster randomized control trial	▪ 20 center-based childcare facilities (10 in intervention, 10 in control)	▪ Evaluate the impact of a cluster randomized trial on the PA levels of 3–5 year old children attending center-based childcare	▪ EPAO (direct observation)		▪ Mean (*SD*) television viewing = 6.90 (21.82) mins/day
			▪ 457 preschoolers; age range = 3–5 years; ~55% male)				
▪ Gunter et al. (2012) [54]	▪ United States	▪ Cross-sectional	▪ 53 home-based childcare facilities	▪ Asses the current status of PA- and nutrition-related policies/practices in home-based childcare facilities to help inform the *Journey to a Healthy Child Care Home* childhood obesity intervention	▪ NAP SACC (proxy-report measure)	▪ Prevalence of screen-viewing policies/practices in centers:	
			▪ 205 preschoolers (age range = 2–5 years)			- 60.4%: *television is turned on every day for at least part of the day*	
						- 58.5%: *children are allowed to watch television/videos or play video games at least once a day*	
						- 20.8%: *children are allowed to use a computer for educational purposes or games at least once a day*	
▪ McWilliams et al. (2009) [55]	▪ United States	▪ Cross-sectional	▪ 96 center-based childcare facilities (only 42 with television data)	▪ Demonstrate how current practices of a large sample of childcare centers compare to best-practice PA guidelines	▪ EPAO (proxy-report measure)		▪ Number of centers engaging in television viewing time (with television present in classroom):
			▪ 66 children/center (median enrollment; where ~50% were 3–5 years)				- 17: ≤ 30 mins/day (or ≤ 0.50 hrs/day)
							- 16: ≥ 31 ≤ 60 mins/day (or ≥ 0.50 ≤ 1 hr/day)
							- 9: > 60 mins/day (or > 1 hr/day)
▪ Natale et al. (2013) [56]	▪ United States	▪ Cross-sectional	▪ 1,140 childcare facilities (842 center-based, 298 home-based)	▪ Explore and differentiate between the PA and nutrition patterns of center- and home-based childcare facilities	▪ Physical Activity Frequency Questionnaire (proxy-report measure)	▪ Television viewing limited to ≤ 60 mins/day (or ≤ 1 hr/day; once a week)	
			▪ Preschoolers’ age range = 3–5 years (~50% male)			▪ Center-based: 474	
						▪ Home-based: 113*	
						▪ Computer use limited to ≤ 60 mins/day (or ≤ 1 hr/day; once a week)	
						▪ Center-based:410	
						▪ Home-based: 186*	
▪ Sisson et al. (2012) [57]	▪ United States	▪ Cross-sectional	▪ 314 center-based childcare facilities	▪ To determine the obesogenic practices of full-day childcare centers	▪ NAP SACC (proxy-report measure)	▪ Prevalence of screen-viewing practices in centers:	
			▪ Preschoolers’ age range = 2–5 years			- 57.4%: *television is rarely/never used*	
▪ Tandon et al. (2011) [58]	▪ United States	▪ Cross-sectional	▪ 6,050 preschoolers (1,900 in home-based childcare; 4,150 in center-based childcare; mean age = 4.37 years [*SE* = 0.01]; 51% male)	▪ Three-fold:	▪ Telephone survey (proxy-report measure)		▪ Mean television viewing:
				1. Assess preschoolers cumulative daily screen time			- Home-based: 1.8 hrs/day^∇^
				2. Measure the contributions of the home and childcare setting to this total			- Center-based: 0.1 hrs/day^∇^
				3. Characterize children most at risk for excessive screen time			▪ Preschoolers in center-based childcare watched significantly less television in comparison to those attending home-based care (*p* < .001)
▪ Taverno Ross et al. (2013) [48]	▪ United States	▪ Multi-component intervention	▪ 16 center-based childcare facilities	▪ Explore the separate influences of “childcare television” vs. “home television” vs. “cumulative television” on preschoolers’ PA and weight	▪ 3-item survey examining the rules, use, and availability of television in childcare (proxy-report measure)	▪ Childcare environment was highly conducive to television viewing	
			▪ 339 preschoolers (mean age = 4.5 years [*SD* = 0.3]; 52.2% males)			▪ Mean (*SD*) of summed scores (i.e., TV availability, rules, use) at baseline = 4.1 (1.8) out of 11 (where a lower score indicates an screen-viewing supportive environment)	
▪ Trost et al. (2009) [59]	▪ United States	▪ Cross-sectional	▪ 297 home-based childcare facilities	▪ Describe nutrition- and PA-related policies/practice in a representative sample of home-based childcare facilities	▪ NAP SACC (proxy-report measure)	▪ Prevalence of screen-viewing policies/practices in centers:	
			▪ Preschoolers’ age range = 2–5 years			- 64.6%: *television is turned on every day for at least part of the day*	
						- 55.1%: *children are allowed to watch television/videos or play video games at least once a day*	
						- 33.2%: *children are allowed to use a computer for educational purposes or games at least once a day*	
▪ Trost et al. (2011) [60]	▪ United States	▪ Quasi-experimental (intervention)	▪ 236 home-based childcare facilities	▪ Determine the impact of a community-based train-the-trainer intervention on the nutrition- and PA-related policies/practice of home-based childcare facilities	▪ NAP SACC (proxy-report measure)	▪ Mean (*SD*) score for television use and viewing: 2.9 (0.8) [out of 4, where a score of 4 = *best practice of never/rarely watching screens*]	
			▪ Preschoolers’ age range = 2–5 years				
▪ Wolfenden et al. (2010) [61]	▪ Australia	▪ Cross-sectional	▪ 261 center-based childcare facilities (112 pre-schools, 149 long-day care settings)	▪ Two-fold:	▪ Telephone survey (proxy-report measure)	▪ 25-30% of centers (preschools and long-day care settings, respectively) provided daily opportunities to engage in screen activities	
			▪ 27 children/pre-school (mean enrolment; age range = 3–5 years)	1. Describe PA-related policies/practices of childcare programs		▪ Policy supports limiting screen recreation	
			▪ 39 children/long-day care setting (mean enrollment; age range = 6 weeks-under 6 years)	2. Ascertain whether characteristics like socio-economic status, remoteness, or size predict these policies and/or practices		- Preschools = 35	
						- Long-day care settings = 69	
▪ Zevenbergen & Logan (2008) [62]	▪ Australia	▪ Cross-sectional	▪ 25 childcare facilities	▪ Determine the amount of access young children had to computers at home and in the childcare setting	▪ Survey (proxy-report measure)	▪ Mean frequency of access to computers in childcare = 1.04^±∇^ (out of 3, where a score of 3 = *frequent*)	
			▪ 150 preschoolers (age range = 4–5 years)			▪ Majority of activities undertaken while using the computer were *educational* games, followed by *non-educational* games	

*Note*: † = personal communication with author (majority of participants were 5 years old with none over 6 years); *SE* = standard error; *SD* = standard deviation; ∇ = value not reported (i.e., study authors did not provide *SD* or presented data was used to extrapolate a value by review author, and as such, no *SD* available); ± = this score may have represented an anomaly (disproportionally high score attributed to one center with a high response rate – when removed, mean frequency = 0.30); * = significant difference; OSRAC-P = Observational System for Recording Physical Activity in Children-Preschool Version; ECERS-P = Early Childhood Environment Rating Scale-Revised Edition; EPAO = Environmental and Policy Assessment and Observation; NAP SACC = Nutrition and Physical Activity Self-Assessment for Child Care; PA = physical activity; MVPA = moderate-to-vigorous physical activity; PAP = physical activity promoting; NPAP = non-physical activity promoting.

### Data synthesis and analysis

Data from included studies were grouped for interpretation based on the outcome variable used to assess screen-viewing within the childcare environment. Where provided, mean rates of daily screen-viewing activities were synthesized for easy comparison across included studies; these values were grouped to create a range (i.e., the lowest and highest means were combined to highlight the extent of screen-viewing among preschoolers in this environment), and thus facilitated analyses. Data comparing screen-viewing in various childcare arrangements (i.e., center- vs. home-based) were also presented when available. Availability for screen-viewing in childcare (i.e., access and opportunities) were reported (and synthesized/integrated where possible) to provide context regarding the opportunities for this behavior to occur during childcare hours.

Using the bioecological theoretical framework, as outlined by Bronfenbrenner and Evans, [63] a number of factors and/or ‘systems’ thought to impact child development were identified (moving from most proximal to the child, to more distal). Subsequently, and in-line with previously published work, [28,29] the correlates of screen-viewing within the childcare environment were divided into the following categories: child demographic factors, staff demographic factors, environmental factors, and social factors. A threshold of three or more studies was used to establish the presence of a potential association between screen-viewing and a particular factor. Extracted correlates were coded using a similar method outlined in other review papers [28,64,65]. In accordance with this model, the *consistency* of association, rather than the strength, was of particular focus.

## Results

### Summary of search efforts and study characteristics

**Figure 1:**
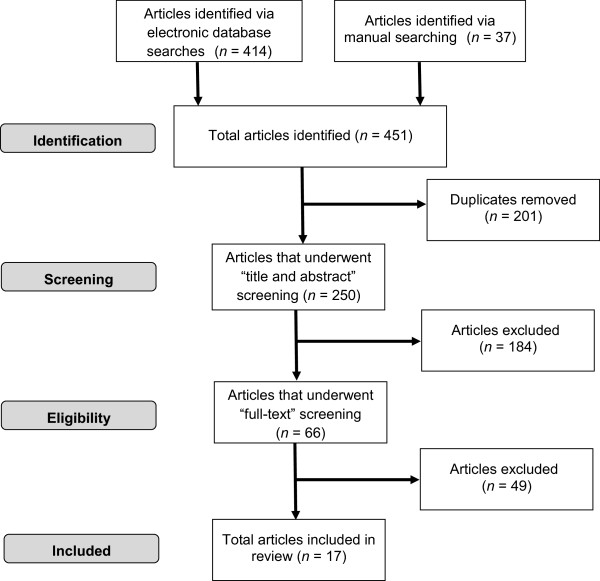
PRISMA flow diagram for identification, screening, eligibility, and inclusion in study.

The electronic database searches yielded 414 relevant articles. An additional 37 articles were retrieved via citation tracking. No new articles were identified through hand searching. After removing duplicates (*n* = 201), 184 articles were excluded following the title and abstract assessment, leaving 66 articles. After reviewing these articles in full for inclusion eligibility, an additional 49 papers were removed. Papers were excluded because: ineligible environment (i.e., not childcare; *n* = 8), absence of a quantitative screen-viewing assessment (*n* = 16), non-primary research (*n =* 12), ineligible age ranges (*n* = 4), failure to differentiate between screen-viewing accumulated at home versus in childcare (*n* = 7), and duplicated/repeated findings (*n* = 2). Consequently, 17 articles were included in this review (see Figure 1 for details on the identification, screening, eligibility, and inclusion process).

Of the incorporated articles, data from 22,039^bc^ participants, across 5,806^b^ childcare facilities and two countries (United States [*n* = 13] [11,43,47,48,50,51,54-60] and Australia [*n* = 4] [52,53,61,62]), were included. Sample sizes ranged from 6 [47] to 8,835^b^[61] preschooler participants, and from 1 [47] to 2,672^b^[11] childcare facilities. Twenty-nine percent of articles examined both types of childcare arrangements (i.e., center- and home-based childcare), while 47% and 24% focused solely on center-based childcare (i.e., private and public programs, preschools) or home-based childcare (i.e., family childcare homes, home daycare), respectively. Articles were published between 2004 [51] and 2014 [53]. Screen-viewing was measured primarily via proxy-report measures, [11,48,50,52,54-62] followed by direct observation [43,47,51,53]. As for *types* of screen mediums; one paper examined video game use, [47] nine focused on television viewing, [11,48,50,53-55,57,58,60] one examined computer use, [62] and five studies explored multiple screen mediums [51,52,56,60,61]. One study refrained from specifying the screen of interest in their paper [43]. See Table 2 for additional details.

### Rate of screen-viewing among preschoolers in childcare

Eight studies reported the rates of daily screen-viewing among preschoolers in childcare (4 via direct observation, and 4 via surveys; Table 2) [11,43,47,50,51,53,55,58]. Screen-viewing ranged from 0.1 to 1.3 hrs/day among preschoolers in center-based childcare, [11,43,50,51,53,55,58] and 1.8 to 2.4 hrs/day among preschoolers in home-based childcare [11,47,50,58]. Three studies examined the differences in screen-viewing based on *type* of childcare arrangement [11,50,58]. In all cases, it was noted that preschoolers in home-based childcare engaged in higher amounts of screen-viewing that those attending center-based facilities. Differences in screen-viewing based on arrangement type were found to be statistically significant in Christakis and Garrison’s (*p* < .001) [50] and Tandon et al.’s (*p* < .001) [58] work, but not in Christakis et al.’s study [11]. Preschoolers in 3^d^ out of 8 studies were found to engage in less than 1-hour of screen-viewing per day in childcare, [43,51,53] 4 out of 8 engaged in 1–2 hours of screen-viewing, [11,47,55,58] and 1 out of 8 engaged in more than 2 hours of screen-viewing [50]. Screen-viewing levels were reported at the center-level, rather than at the individual child-level (save Bacigalupa’s work [47]). Few studies commented on the context/purpose behind participants’ decision to engage in screen-viewing activities while in childcare [50,54,59,62]. Only two papers identified which percentage of screen-viewing behavior was considered *active* (i.e., light physical activity or moderate-to-vigorous physical activity [MVPA]) versus *sedentary*[43,51].

### Context of screen-viewing activities

Two of the 17 studies specified that the majority of preschoolers’ screen-viewing behaviors in care were sedentary, [43,51] with very little physical activity being accumulated while engaging in such activities. Approximately 27% of the studies also noted the purpose behind preschoolers’ participation in screen-viewing activities while in childcare, [50,54,59,62] with the most prevalent being education- and entertainment-related.

### Correlates of screen-viewing in childcare

#### Child demographic factors

Only one study commented on the relationship between preschooler sex and screen-viewing; [47] thus, no association to screen-viewing in childcare was reported (i.e., less than 3 studies). See Table 3 for additional details.

**Table 3:** Correlates of screen-viewing among preschoolers in childcare

**Factor type**	**Factor**	**Association**		**Strength of consistency supporting the association**
		**+**	**-**	
**Child demographic factors**	Sex	[47] {H}		Inconclusive
**Staff demographic factors**	High level of education		[11] {C}	Strong
			[50] {C}	
			[58] {C}	
	High volume of staff/center		[C] [11]	Inconclusive
**Environmental factors**	Daily hours of operation	[11] {H/C}		Inconclusive
	Type of childcare arrangement	[11] {H},		Strong
		[50] {H},		
		[56] {H},		
		[58] {H},,		
	Provision of after-school care	[11] {C}		Inconclusive
	Open practices/policies re: screen use	[48] {C}		Inconclusive
**Social factors**	Low SES neighborhood	[11] {C}		Inconclusive

*Note*. H = home-based childcare; C = center-based childcare; H/C = both childcare arrangement types; SES = socio-economic status; inconclusive = fewer than 3 studies examined the variable (therefore, no conclusions could be drawn); strong consistency = 75-100% of studies examining the factor support the association.

#### Staff demographic factors

A negative association was identified in studies reporting a relationship between screen-viewing and high levels of staff education (3 negative associations/3, 100%) [11,50,58]. No association between screen-viewing and high staff volume was ascertained (i.e., less than 3 studies). See Table 3.

#### Environmental factors

A positive association was highlighted between type of childcare arrangement (notably, home-based in comparison to center-based facilities) and increased screen-viewing among participants (4 positive associations/4, 100%) [11,50,56,58]. Other correlates, such as the provision of after-school care, [11] daily hours of operation, and open practices/policies regarding screen-use in childcare, [48] were reported in less than three studies, and therefore no overall associations were determined (Table 3).

#### Social factors

Only one study reported a relationship between screen-viewing in childcare facilities in low SES neighborhoods (Table 3); [50] the association was identified as inconclusive (less than 3 studies).

### Availability of screen-viewing opportunities in childcare

Nine studies reported on the availability of screen-based activities (i.e., access to/opportunities for screen-viewing) within the childcare environment (Table 2) [48,52,54,56,57,59-62]. While many of these studies utilized a slightly different method of assessing screen availability and access (i.e., screen use policies and practices [*n* = 6], access to screens and/or activities while in care [*n* = 3]), this information provides credence to the high rates of screen-viewing in childcare facilities.

When analyzing the childcare environment as a whole, Trost and colleagues found that the participating home-based childcare facilities (*n* = 236) were, for the most part meeting (but not exceeding) their respective standards of rarely/never showing television or videos [60]. In contrast, Taverno Ross et al. found center-based childcare facilities were highly conducive to screen-viewing (based on high availability and frequency of screen-use) [48]. Zevenbergen and Logan also reported that preschoolers in the childcare setting had fairly regular access to computers [62].

With regard to specific policies and practices concerning screen-use in childcare, projects by Trost et al. (*n* = 294 facilities) [59] and Gunter et al. (*n* = 53 facilities) [54] reported that the majority of home-based facilities had the television turned on every day (for at least a portion of the day; 64.6% and 60.4%, respectively) and also permitted children to play video games and/or watch television at least once a day (55.1% and 58.5%, respectively); computer use was also permitted in a number of facilities across both studies. While Sisson et al. found approximately 60% of participating centers rarely/never permitted children to watch television [57], Natale and colleagues’ work indicated 474 center- and 113 home-based childcare facilities restricted television-viewing to 1-hour per day, and that 410 centre- and 186-home-based facilities limited their computer use to this same time restriction [56]. Over half the centers in Wolfenden and colleagues’ paper [61] and 45% of those in Finch et al.’s [52] paper had policies in place limiting screen-use during care hours (but did not provide specifics). While policies and practices to curtail this behavior are evident across some studies (*n* = 4), ease of access and opportunities to engage in screen-viewing activities in childcare are prevalent (*n* = 5). Consideration of both screen-viewing policies and accessibility is important to examine in order to understand the screen-time rates reported previously.

## Discussion

This systematic review aimed to report the frequency of screen-viewing among preschoolers in childcare. As a secondary objective, this review explored correlates of screen-viewing within this setting.

### Screen-viewing among preschoolers in childcare

The chief finding of this review suggests preschoolers, in general, participate in somewhat high levels of daily screen-viewing while in childcare, although substantial variation exists. Of the papers that reported rates of screen-viewing, [11,43,47,50,51,53,55,58] levels of screen-time ranged from 0.1 to 1.3 hrs/day and 1.8 to 2.4 hrs/day among preschoolers in center- and home-based childcare, respectively. While considerable variation across these studies is evident (with two studies reporting minimal screen-viewing levels among their center-based samples), [43,58] the results of this review suggest participants in five studies are exceeding Canadian/Australian [26,27] (i.e., 1 hour/day limit) guidelines, [11,47,50,53,55] and preschoolers in one study are surpassing the American [25] screen-viewing guidelines (i.e., 2 hour/day limit) [58]. These numbers are concerning, particularly due to the fact that these guidelines refer to total *daily* screen-viewing; because this paper focused solely on screen-viewing among preschoolers in childcare, it is possible that this population could engage in additional screen-viewing outside of care. In fact, recent work by Tandon et al. [58] which examined screen-viewing during and outside childcare hours, found that children in center- and home-based childcare accumulated an additional 3.1 and 3.8 hours of screen-viewing while at home, respectively. As per the recent finding that screen-viewing in excess of 2 hours is associated with poorer psychosocial and physical health among children, [66] action is required to decrease the amount of time this population spends engaging in this behavior. Interestingly, Brown et al. [43] and Dowda et al. [51] found a small percentage of preschoolers’ screen-viewing time was actually spent in light physical activity or MVPA (specific activities not identified in-text). Accordingly, these findings highlighted that not all screen-viewing activities require inactivity, but that perhaps the childcare environment could shift the way in which screens are used in order to include more activity.

### Screen-viewing based on medium type

Levels of television viewing were assessed most often (i.e., assessed in 82% of the studies and was the single screen of focus in 53% of cases). One reason explicating this finding could be that one television can entertain many children with little input from staff, rather than other types of screens (i.e., computers, tablets) which entertain only a few children at a time and require more monitoring (i.e., to ensure sharing of the devices across children) and/or assistance from staff. It is also possible that, measurement of television only is a result of the age of the studies included, as computer use, iPads, etc. might have only recently become popular and available in these facilities. However, by focusing solely on one type of screen medium (rather than all mediums accessible in childcare), researchers may be underestimating preschoolers’ total screen-time in care, and rendering it difficult to properly address this issue. While there is research to support that young children spend the majority of their screen-time watching television (and very little time engaged in other screen-based activities, like computers) within the home environment, [67] it would be interesting to explore whether similar trends exist in the childcare environment; it would also be interesting to explore whether said trends are likely to continue in light of the growing popularity of other screens and as new research on this topic emerges. Consequently, future research efforts should examine the differences in screen-viewing among preschoolers in care based on all medium types accessible by this group. This will not only capture a clearer picture of preschoolers’ screen preferences, but will also assist in determining whether environmental modifications and/or the introduction (or ratification) of policies that target specific screen types are warranted.

### Correlates to Preschoolers’ screen-viewing levels in childcare

Building on Christakis et al.’s previous work (which administered surveys to childcare staff), [11] this review synthesized the literature regarding influential factors associated with preschoolers’ television viewing [only] in childcare. Only 2 out of 8 variables were identified as correlates to preschoolers’ screen-viewing in childcare. High staff education was found to have a negative association with participants’ screen-viewing levels; [11,50,58] children spent less time participating in screen-viewing activities when staff were more highly educated. Similar findings corresponding to other health behaviors have been highlighted in the literature; more favorable nutrition and physical activity outcomes have been observed among preschoolers cared for by more highly educated and trained staff [68,69]. Due to the important role childcare staff play in promoting and modeling both negative and positive health behaviors, [33,68] precedence should be given to providing training and education related to screen-viewing (and its associated health implications) to these key individuals.

The second potential correlate to preschoolers’ screen-viewing in childcare, the *type* of childcare arrangement, demonstrated a positive association to this behavior. Notably, children attending home-based childcare may be more prone to screen-viewing activities. In fact, *all* studies examining screen-viewing among preschoolers in home-based childcare [11,47,50,58] reported their participants as surpassing Canada’s recommended 1-hour guideline [26]. While comparing levels of television viewing across both arrangements, Christakis and Garrison found that preschoolers enrolled in home-based childcare accumulated ~2 hours of screen-time over that which was accumulated by those attending centre-based programs [50]. Even more disturbing is the fact that children in home-based facilities have been found to accumulate approximately 5.6 hours of *daily* screen time (3.8 hours at home and 1.8. hours in childcare), in comparison to those in center-based care (3.1 hours at home and 0.1 hours in childcare) [58]. While it has been found that children who attend these settings are likely to be heavier than those in center-based childcare, [70,71] reinforcing the need to target preschoolers in this particular care setting. There are multiple reasons that may explain this finding. Firstly, it is possible that because home-based facilities care for children of various ages (in comparison to centers that have children separated based on age/developmental stage [72]), screen-viewing may be viewed as an appropriate activity in which *all* children can partake. Secondly, given that these types of facilities vary considerably with regard to layout and structure, [58] it is not surprising that a lack of appropriate indoor play space may restrict active behaviors and thus support prolonged periods of sitting and screen-use [59]. Thirdly, because there is only one provider present in this particular type of childcare arrangement, it may prove more challenging to carry out certain tasks, such as meal preparation; consequently, screen-viewing may serve as an ideal ‘babysitter’ during such instances. Lastly, home-based childcare facilities tend to be less regulated than center-based programs; [73,74] therefore, in addition to lacking policies regarding screen-time/use, the childcare providers do not require any formal education to run this business out of their private homes.

### Availability of screen-viewing opportunities during childcare hours

The link between access to screen sources and high screen-viewing levels has been well-established in the literature; [16,28] subsequently, it was important to garner a deeper understanding of the opportunities for and accessibility to screen activities for preschoolers during childcare hours (as described by nine of the included studies) [48,52,54,56,57,59-62]. The results of this review found screens and related activities (namely television followed by computers) were accessible to preschoolers in childcare. Consequently, these participants were at-risk for accumulating higher levels of screen-viewing. Nevertheless, it is important to note that the presence of screen-use policies in childcare was highlighted in over half of the studies in this review that spoke to the availability screen-viewing in childcare; [48,52,54,56,59] therefore suggesting that, in many instances, mechanisms are in place to help minimize screen-viewing in childcare. Of particular interest, Trost’s group reported the majority of participating home facilities were meeting the Nutrition and Physical Activity Self-Assessment for Child Care’s (NAP SACC) proposed screen-viewing standards (i.e., rarely/never showing television or videos during care hours) [60]. While this discovery suggests opportunities to engage in related activities may be limited in care, it somewhat contradicts the apparent high levels of screen-viewing in home childcare observed in this review [11,50,58]. One possible explanation for this score could be that the NAP SACC tool is administered to staff as a self-assessment, and may in turn be subject to bias given the nature of the questions asked (e.g., amount of screen-time permitted per week; presence of written policies on screen time; discussing with children what they are viewing when screen time is offered; screen time used a reward; location of screens in childcare facilities) [75]. It is also possible that, despite the existence of screen-viewing policies and rules, there is no guarantee that these policies are being practiced nor enforced during childcare hours. Lastly, in light of the existing variation across the different screen-viewing policies presented in-text, much variability in related outcomes among young children may result; as such, further research is necessary to not only explore the emerging screen-related behaviors within these facilities, but also the implementation and enforcement of associated policies.

### Limitations

Due to the limited available research, all study designs and quality of evidence were eligible for inclusion in this review. As a result, it was not possible to review only the highest quality of evidence; a factor that may have affected the overall strength of the current paper’s findings. Secondly, it was difficult to establish casual relationships between screen-viewing and the identified correlates as many of the included studies were cross-sectional. Thirdly, of the studies that reported rates of screen-viewing, there was a lack of consistency in the manner in which these papers presented their findings. For example, some focused solely on one medium while others provided a combined measure, thus rendering it difficult to extract findings and compare levels of screen-viewing based on screen *type*. Lastly, it proved difficult to appropriately synthesize some of the included studies’ findings (e.g., rate of screen-viewing) because it was not possible in all cases to determine a proper range or upper limit value.

## Conclusion

This paper underscores the infancy of this research as only 17 studies, all published in the last decade, explored the prevalence of screen-time in childcare as well as related correlates. This is the first systematic review to identify the frequency of, and opportunities for, screen-viewing among preschoolers in this unique environment. The results of this review propose that, despite some variability, preschoolers appear to engage in somewhat high levels of screen-viewing while in childcare, particularly within home-based facilities. This paper also highlights the conduciveness of the childcare environment with regard to screen-viewing among preschoolers. The apparent association between increased staff education levels and decreased screen-viewing suggest additional training and education on screen-viewing and sedentary behaviors should be viewed as a priority. Further investigations are needed not only to establish a solid understanding of preschoolers’ habitual screen-viewing behaviors while in childcare, but to modify the childcare environment (and the policies/curriculum therein) in a way that best minimizes screen-related activities during childcare hours as well.

## Endnotes

^a^Contact author for full search strategy details.

^b^These figures represent *best* estimates. Seven studies did not present the total number of preschooler participants, [11,50,52,56,57,59,60] while another failed to report the number of childcare facilities involved. [54] Consequently, these values may represent conservative underestimations.

^c^Two studies provided a mean number of children included; [55,61] however, did not provide a total, nor distinguish between the proportion of preschoolers and the proportion of infants/toddlers. Therefore, an extrapolation of the total number of participating children was estimated.

^d^Two papers did not present and upper limit to the screen-viewing levels observed in their studies (i.e., great than 60 minutes) [51,55]. This range may represent an underestimation of screen-time in children.

## Abbreviations

MVPA: Moderate-to-vigorous physical activity; NAP SACC: Nutrition and physical activity self-assessment for Child care.

## Competing interests

The author declares she has no competing interests.

## Author’s contributions

LMV was the sole author of this paper. As a result, she conceptualized the review objective; carried out all data collection, syntheses, and analyses; drafted the manuscript (and revised subsequent drafts); and, approved the final version of the paper.

## Author’s information

LMV is a PhD candidate at Western University in Health and Rehabilitation Sciences (field: Health Promotion). She is currently a research coordinator at the Child Health and Physical Activity Lab at Western.

## Pre-publication history

The pre-publication history for this paper can be accessed here:

http://www.biomedcentral.com/1471-2431/14/205/prepub
